# Fractional Calculus as a Tool for Modeling Electrical Relaxation Phenomena in Polymers

**DOI:** 10.3390/polym17131726

**Published:** 2025-06-20

**Authors:** Flor Y. Rentería-Baltiérrez, Jesús G. Puente-Córdova, Nasser Mohamed-Noriega, Juan Luna-Martínez

**Affiliations:** 1 Facultad de Ciencias Químicas, Universidad Autónoma de Nuevo León, Av. Universidad s/n, San Nicolás de los Garza 66455, Mexico; flor.renteriabltz@uanl.edu.mx; 2 Facultad de Ingeniería Mecánica y Eléctrica, Universidad Autónoma de Nuevo León, Av. Universidad s/n, San Nicolás de los Garza 66455, Mexico; juan.lunamrt@uanl.edu.mx

**Keywords:** fractional calculus, relaxation, dielectric, interfacial polarization, electrical fractional model

## Abstract

The dielectric relaxation behavior of polymeric materials is critical to their performance in electronic, insulating, and energy storage applications. This study presents an electrical fractional model (EFM) based on fractional calculus and the complex electric modulus ($M^{*}=M^{\prime }+iM^{\prime \prime }$) formalism to simultaneously describe two key relaxation phenomena: α-relaxation and interfacial polarization (Maxwell–Wagner–Sillars effect). The model incorporates fractional elements (cap-resistors) into a modified Debye equivalent circuit to capture polymer dynamics and energy dissipation. Fractional differential equations are derived, with fractional orders taking values between 0 and 1; the frequency and temperature responses are analyzed using Fourier transform. Two temperature-dependent behaviors are considered: the Matsuoka model, applied to α-relaxation near the glass transition, and an Arrhenius-type equation, used to describe interfacial polarization associated with thermally activated charge transport. The proposed model is validated using literature data for amorphous polymers, polyetherimide (PEI), polyvinyl chloride (PVC), and polyvinyl butyral (PVB), successfully fitting dielectric spectra and extracting meaningful physical parameters. The results demonstrate that the EFM is a robust and versatile tool for modeling complex dielectric relaxation in polymeric systems, offering improved interpretability over classical integer-order models. This approach enhances understanding of coupled relaxation mechanisms and may support the design of advanced polymer-based materials with tailored dielectric properties.

## 1. Introduction

Nowadays, polymeric materials play a very important role in various fields of science and engineering, particularly in the electrical and electronic sectors, where they perform as organic insulators, dielectrics, and semiconductors [1,2,3]. Moreover, new methods have made it possible to even synthesize conductive polymers; when combined with inorganic micro- and nanoparticles, these materials give rise to more complex polymeric systems, resulting in a diversity of materials with a broader range of properties. Nevertheless, this new range of properties is defined by the conductivity, permittivity, and dielectric strength; in addition to the physical and chemical aging, which are responsible for the decay of the electrical properties over time [4,5]. In this sense, it is essential to carry out relaxation phenomena studies to optimize the desired properties for a specific application.

A dielectric relaxation process is associated with a specific molecular reconfiguration which generates a new structural equilibrium with lower energy since an amorphous phase is far from the thermodynamic equilibrium [6,7,8]. Therefore, the measurement of the α-relaxation (or main relaxation), in the vicinity of the glass transition region, is essential to understand the molecular mobility. From the electrical perspective, the glass transition of polymers is a complex phenomenon, involving the simultaneous and cooperative motion of the electrical dipoles of the polymer when subjected to an external electrical field; this phenomenon not only depends on the structure and morphology of the polymer, but it is also temperature-dependent and proceeds with a rate that increases with temperature. Thermally stimulated discharge current (TSDC), dynamic mechanical analysis (DMA), and dynamic electrical analysis (DEA) are the most used characterization techniques employed to study the structural relaxations in polymers since they can follow the evolution of the properties during the relaxation processes. Nevertheless, the use of DEA is preferred due to its higher sensitivity to molecular motions of chemical groups with a dipolar moment and its larger range of working frequencies and temperatures.

Fractional calculus is the branch of mathematics that deals with the generalization of integrals and derivatives of arbitrary orders, and it has been successfully used as a powerful mathematical tool to model the complex relative permittivity ε_r_* of polymeric systems [7,9], allowing for the determination of the correlation between the molecular mobility and the experimental data obtained by DEA. The application of fractional calculus to study the dielectric relaxation phenomena of polymers has involved using the “cap-resistor”, a fractional element proposed by Reyes-Melo [10], which intimately combines the dielectric behavior (capacitor element) with the insulator behavior (resistor element) by a differential operator of fractional order. This fractional element is analogous to the Scott–Blair or “spring-pot” element used in linear viscoelasticity [11,12]. The idea is to replace the resistor element on the classical Debye model with two cap-resistors; the modified Debye model is called the “fractional Debye model” (FDM).

In previous works with polyvinyl butyral (PVB) [13] and poly(ethylene-2,6-napthalene dicarboxylate) (PEN) [7], the fractional order parameters of the FDM have been related to the cooperative molecular mobility during the α-relaxation; it was found that the molecular mobility related to α-relaxation at temperatures above the glass transition temperature (*Tg*) differs from that observed below *Tg*. Additionally, Luo and Chen [14] used the cap-resistor element to work out an electrical version of the Maxwell model to fit the dielectric data of liquid crystal cells loaded with Pd nanoparticles. More recently, Meng [15] studied the non-linear behavior of ferroelectric polymer composites with the aid of fractional calculus, using the spring-pot and the cap-resistor elements for the mechanical and dielectric behavior, respectively.

In addition to molecular mobility, when a polymer is subjected to an external electric field, extrinsic and intrinsic carriers can produce an accumulation of electrical charges on the surface or the bulk of the polymer. This phenomenon is called “space charge”, and it is considered one of the main causes of accelerated aging; thus, studying its magnitude, its spatial distribution inside the polymer, and its origin is crucial. In DEA measurements, it has been observed that, above *Tg* and at low frequencies (10^−3^–10^2^ Hz), the generation of space charge in polymers is affected by ionic conductivity [16,17]; however, these observations were obtained using ε_r_*, which obscured the experimental results. An alternative approach is the use of the complex electric modulus M* [18,19,20], which is analogous to the complex elastic modulus E* used in linear viscoelasticity; both phenomena are considered to be “true relaxations”.

Several studies can be found in the literature on this topic, with polyethylene (PE) being the most studied polymer, due to its simple macromolecular structure and large-scale industrial application [21,22,23]. Furthermore, Mudarra et al. investigated the space charge relaxation of amorphous polymethyl methacrylate (PMMA) [17,24]. Using the TSDC technique, they observed a strong dependence between the space charge, the charge carriers injected into the polymer, and the nature of the traps. Additionally, studies on polyetherimides (PEIs), polyetheretherketone (PEEK), and PMMA have also been performed using DEA [16,17,25], but despite the great contributions to the measurement of the space charge and interfacial polarization in polymeric systems, interpreting experimental data remains a challenge, which may be better interpreted through fractional order operators. The main objective of this work is to develop and validate an electrical fractional model (EFM) capable of simultaneously describing two key dielectric relaxation mechanisms in polymers: the primary α-relaxation and the interfacial polarization known as the Maxwell–Wagner–Sillars (MWS) effect. This is achieved by using the electric modulus formalism in combination with fractional calculus, providing a robust analytical framework that links physical relaxation phenomena with measurable dielectric behavior. The novelty of this approach lies in the use of fractional differential equations to integrate two relaxation processes within a single model, enhancing both the interpretability of parameters and the fitting accuracy compared to classical models. Unlike traditional empirical functions, the EFM offers a direct relationship between fractional orders and molecular mobility.

## 2. The Cap-Resistor, the Fractional Debye Model, and the Electrical Fractional Model

### 2.1. Fractional Calculus and Dielectric Models

In recent years, there has been an important advance in the use of fractional order operators (derivatives and integrals) to describe mechanical, electrical, thermal, and magnetic phenomena [26,27,28]. Particularly, for the analysis of the dielectric properties of materials like polymers, the models have been built with equivalent electrical circuits based on resistors and capacitors to account for the insulation and dielectric nature of these materials. The classical Debye model describes dielectric relaxation in ideally homogeneous materials using integer-order derivatives. However, many real materials exhibit non-ideal relaxation processes that cannot be adequately described by this approach.

Moreover, classical models like the Debye model consider only a single relaxation process, which is far from what has been observed experimentally in polymeric systems as a result of the polydispersity of the polymeric chains. Furthermore, it has been reported that molecular weight distribution is proportional to the relaxation times distribution [29]; therefore, the experimental plot for the complex permittivity, particularly the Cole–Cole plot, is an asymmetric curve. Since the Debye Model does not properly fit the experimental data, empirical models have been proposed, like the Cole–Cole, Cole–Davidson, and Havriliak–Negami, with the last one considered the most used. Nevertheless, little research has been conducted with models using fractional calculus for the study of the relaxation phenomena based on the electric modulus.

**Figure 1:**
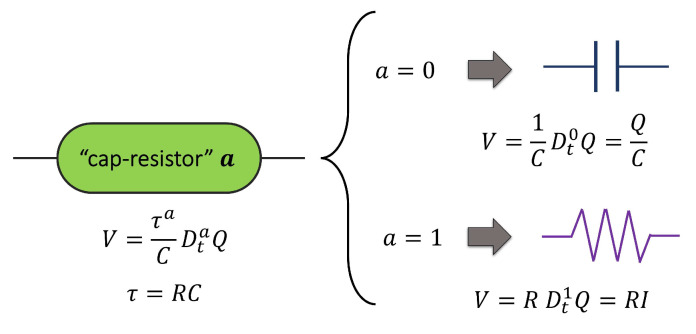
The cap-resistor element. When the fractional order a = 0, the behavior corresponds to the capacitor element (Faraday’s law), and when a = 1, the behavior corresponds to the resistor element (Ohm’s law).

To develop an electrical model that can accurately describe experimental results, a fractional element called cap-resistor was used (see Figure 1); this element represents an intermediate behavior between a resistor and a capacitor. The constitutive equation of the cap-resistor is defined as Equation (1).

(1) $$V=\frac{{\left(RC\right)}^{a}}{C}D_{t}^{a}Q=\frac{\tau^{a}}{C}D_{t}^{a}Q$$

where V is the voltage, R is the resistance, C is the capacitance, Q is the electric charge, and $D_{t}^{a}$ is the fractional derivative of order a. The parameter τ represents the relaxation time, related to the time required by electrical dipoles for a complete reconfiguration to a new structural equilibrium condition.

The fractional order takes values between 0 and 1; a pure dielectric behavior is observed when the fractional order of the cap-resistor element is 0, while a value of 1 represents a pure resistive behavior. The fractional derivative operator is expressed using the Riemann–Liouville definition [30,31], Equation (2), which has a power-law kernel:

(2) $$D_{t}^{a}Q=\frac{1}{\Gamma \left(1-a\right)}\frac{d}{dt}\int_{0}^{t}{\left(t-\xi \right)}^{-a}Q\left(\xi \right)d\xi$$

Here, 0<a<1, and Γ(·) is the gamma function. Although there is currently no accepted physical meaning for a fractional derivative, some authors have proposed that the fractional order can be associated with the rate of partial energy stored or dissipated [32,33,34]. Regarding polymer science, Reyes-Melo et al. developed a mechanical and dielectric model that accounts for three relaxation phenomena in PEN, establishing a relationship between the fractional order and the degree of crystallinity [7]. Rentería-Baltiérrez et al. reported that the fractional order depends on the molecular weight of the polymer PVB and is also sensitive to interactions between polymer chains and iron oxide nanoparticles [13]. More recently, Miranda-Valdez et al. investigated the viscoelastic behavior of aqueous methylcellulose systems, finding that the fractional orders are associated with molecular mobility during heating–cooling cycles and the thermogelation process [35].

**Figure 2:**
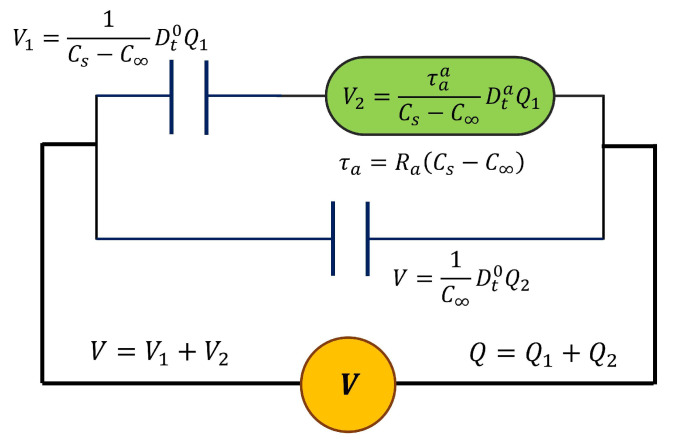
The fractional Debye model with one cap-resistor.

In order to obtain a symmetric response and consider the contribution of interfacial polarization or Maxwell–Wagner–Sillars (MWS) relaxation, we replaced the resistance on the Debye Model with a single cap-resistor and obtained a fractional Debye model (FDM), which is presented in Figure 2. From the constitutive equations that are presented in Figure 2, the differential equation of fractional order is obtained using Equation (3).

(3) $$V=\frac{Q-C_{\infty }V}{C_{s}-C_{\infty }}+\frac{\tau^{a}}{C_{s}-C_{\infty }}D_{t}^{a}\left[Q-C_{\infty }V\right]$$

Here, the cap-resistor characterizes the dielectric response in the region around dielectric relaxation, and the two capacitor elements represent the storage dielectric response. $C_{s}$ is the capacitance at low frequencies or high temperatures, and $C_{\infty }$ is the capacitance at high frequencies or low temperatures.

Subsequently, the frequency response is obtained by applying the Fourier transform of a fractional differential operator $D_{t}^{a}f(t)$ and is written as the product of ${\left(i\omega \right)}^{a}$ by the Fourier transform of the function f(t) [36]. Finally, a mathematical expression for the complex relative permittivity is obtained (Equation (4)), where the real part is associated with the storage of electric charge, while the imaginary part is associated with the energy dissipation.

(4) $$\varepsilon_{r}^{*}=\varepsilon_{r}^{\prime }-i\varepsilon_{r}^{\prime \prime }=\frac{\varepsilon_{rs}+\varepsilon_{r\infty }{\left(i\omega \tau_{a}\right)}^{a}}{1+{\left(i\omega \tau_{a}\right)}^{a}}$$

Here, $\varepsilon_{rs}$ is the relative permittivity at low frequencies or high temperatures, $\varepsilon_{r\infty }$ is the relative permittivity at high frequencies or low temperatures, and $\tau_{a}$ is defined in this work as the characteristic time for an electrical relaxation process. In polymers, the relaxation time is temperature-dependent and, under isothermal conditions, can be considered as constant. The electric modulus M* is mathematically defined as the inverse of the relative permittivity $\varepsilon_{r}^{*}$ (Equation (5)), and from a physical point of view, it is related to the relaxation of the electric field when the electric displacement remains constant [19,37]. Then, using Equations (4) and (5), an expression for $M^{*}$ is obtained that corresponds to the FDM (Equation (6)).

(5) $$M^{*}=M^{\prime }+iM^{\prime \prime }=\frac{1}{\varepsilon_{r}^{*}}=\frac{1}{\varepsilon_{r}^{\prime }-i\varepsilon_{r}^{\prime \prime }}=\frac{\varepsilon_{r}^{\prime }}{{\varepsilon_{r}^{\prime }}^{2}+{\varepsilon_{r}^{\prime \prime }}^{2}}+i\frac{\varepsilon_{r}^{\prime \prime }}{{\varepsilon_{r}^{\prime }}^{2}+{\varepsilon_{r}^{\prime \prime }}^{2}}$$

(6) $$M^{*}=M_{\infty }M_{s}\frac{1+{\left(i\omega \tau_{a}\right)}^{a}}{M_{\infty }+{M_{s}\left(i\omega \tau_{a}\right)}^{a}}$$

Here, ${M_{\infty }=1/\varepsilon }_{r\infty }$ is the electric modulus at high frequencies, and ${M_{s}=1/\varepsilon }_{rs}$ is the electric modulus at low frequencies. Equation (6) is then separated into the real and imaginary components:

(7) $$M^{\prime }=M_{\infty }M_{s}\frac{M_{\infty }+\left(M_{s}+M_{\infty }\right)\left[\cos\left(a\frac{\pi }{2}\right){\left(\omega \tau_{a}\right)}^{a}\right]+M_{s}{\left(\omega \tau_{a}\right)}^{2a}}{M_{\infty }^{2}+2M_{s}M_{\infty }\left[\cos\left(a\frac{\pi }{2}\right){\left(\omega \tau_{a}\right)}^{a}\right]+M_{s}^{2}{\left(\omega \tau_{a}\right)}^{2a}}$$

(8) $$M^{\prime \prime }=M_{\infty }M_{s}\frac{\left(M_{\infty }-M_{s}\right)\left[\sin\left(a\frac{\pi }{2}\right){\left(\omega \tau_{a}\right)}^{a}\right]}{M_{\infty }^{2}+2M_{s}M_{\infty }\left[\cos\left(a\frac{\pi }{2}\right){\left(\omega \tau_{a}\right)}^{a}\right]+M_{s}^{2}{\left(\omega \tau_{a}\right)}^{2a}}$$

**Figure 3:**
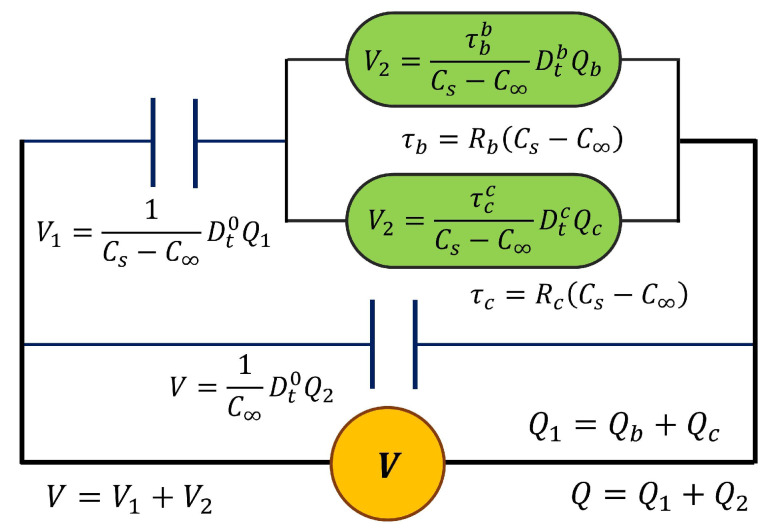
The fractional Debye model (FDM2) modified with two cap-resistors.

When a=1, it is possible to recover the classical expressions for Debye’s model. The loss factor is calculated as $\tan\delta =M^{\prime \prime }/M^{\prime }$. However, this model does not represent the typical response for the main relaxation or α-relaxation of polymeric materials (electrical manifestation of the glass transition); the Cole–Cole plot in the complex plane is asymmetrical. In order to obtain an asymmetric response, the resistance is replaced with two cap-resistors; see Figure 3. This model is the fractional Debye model (FDM2) modified with two cap-resistors.

The fractional differential equation (Equation (9)) is obtained considering the constitutive equations from Figure 3.

(9) $$Q=C_{\infty }V+\left(C_{s}-C_{\infty }\right)\left[\tau_{b}^{-b}D_{t}^{-b}\left(V-\frac{Q-C_{\infty }V}{C_{s}-C_{\infty }}\right)+\tau_{c}^{-c}D_{t}^{-c}\left(V-\frac{Q-C_{\infty }V}{C_{s}-C_{\infty }}\right)\right]$$

where 0<b,c<1 and b<c. The fractional order b corresponds to the molecular mobility at high frequencies or low temperatures, while the fractional order c is related to the molecular mobility at low frequencies or high temperatures, around the α-relaxation. The relaxation times $\tau_{b}$ and $\tau_{c}$ correspond to the cap-resistors b and c, respectively. Using the Fourier transform, the equation for the complex relative permittivity is obtained using Equation (10).

(10) $$\varepsilon_{r}^{*}=\varepsilon_{r}^{\prime }-i\varepsilon_{r}^{\prime \prime }=\frac{\varepsilon_{r\infty }+\varepsilon_{rs}\left[{\left(i\omega \tau_{b}\right)}^{-b}+{\left(i\omega \tau_{c}\right)}^{-c}\right]}{1+\left[{\left(i\omega \tau_{b}\right)}^{-b}+{\left(i\omega \tau_{c}\right)}^{-c}\right]}$$

Then, the complex electric modulus for FDM2 is calculated as follows:

(11) $$M^{*}=M_{\infty }M_{s}\frac{1+\left[{\left(j\omega \tau_{b}\right)}^{-b}+{\left(j\omega \tau_{c}\right)}^{-c}\right]}{M_{s}+M_{\infty }\left[{\left(j\omega \tau_{b}\right)}^{-b}+{\left(j\omega \tau_{c}\right)}^{-c}\right]}$$

Equation (11) can be split into the real and imaginary parts as follows:

(12) $$M\prime =M_{\infty }M_{s}\frac{M_{s}+A_{1}\left(M_{s}+M_{\infty }\right)+M_{\infty }\left(A_{1}^{2}+A_{2}^{2}\right)}{{\left(M_{s}+M_{\infty }A_{1}\right)}^{2}+{\left(M_{\infty }A_{2}\right)}^{2}}$$

(13) $$M^{\prime \prime }=M_{\infty }M_{s}\frac{\left(M_{\infty }-M_{s}\right)A_{2}}{{\left(M_{s}+M_{\infty }A_{1}\right)}^{2}+{\left(M_{\infty }A_{2}\right)}^{2}}$$

(14) $$A_{1}=cos\left(b\frac{\pi }{2}\right){\left(\omega \tau_{b}\right)}^{-b}+cos\left(c\frac{\pi }{2}\right){\left(\omega \tau_{c}\right)}^{-c}\;A_{2}=sin\left(b\frac{\pi }{2}\right){\left(\omega \tau_{b}\right)}^{-b}+sin\left(c\frac{\pi }{2}\right){\left(\omega \tau_{c}\right)}^{-c}$$

Using the equivalent circuits of Figure 2 and Figure 3, it is possible to obtain a mathematical expression to describe two relaxation phenomena, the electrical fractional model (EFM). $M^{*}$ is determined using Equations (15) and (16). The first term involves a single cap-resistor, a, associated with the MWS relaxation or interfacial polarization. The second possesses two cap-resistors, b and c, and is associated with the α-relaxation.

(15) $$M^{*}=M_{MWS}^{*}+M_{\alpha }^{*}$$

(16) $$M^{*}=\left[M_{1\infty }M_{1s}\frac{1+{\left(i\omega \tau_{a}\right)}^{a}}{M_{1\infty }+{M_{1s}\left(i\omega \tau_{a}\right)}^{a}}\right]+\left[M_{2\infty }M_{2s}\frac{1+\left[{\left(j\omega \tau_{b}\right)}^{-b}+{\left(j\omega \tau_{c}\right)}^{-c}\right]}{M_{2s}+M_{2\infty }\left[{\left(j\omega \tau_{b}\right)}^{-b}+{\left(j\omega \tau_{c}\right)}^{-c}\right]}\right]$$

### 2.2. Temperature Dependence of Relaxation Times

The response of $M^{*}$ in the temperature domain (under isochronal conditions) can be analyzed through the temperature dependence of molecular relaxation times associated with the electrical behavior of polymeric materials. From an experimental point of view, Arrhenius-type diagrams (τ vs. 1/T) are constructed to represent relaxation phenomena in a polymer. These are known as relaxation maps, functioning as a molecular mobility fingerprint for each polymer [13,38]. Dielectric spectroscopy is the most widely used technique for this purpose, covering a broad frequency range (from 10^−3^ Hz to 10^9^ Hz). In contrast, mechanical spectroscopy has a more limited frequency window (f < 10^2^ Hz). Moreover, dielectric and mechanical relaxation maps are not identical, due to the different nature of stimuli applied to the polymer (body forces vs. surface forces).

A typical relaxation map for a polymer may exhibit three main relaxation processes: the α-relaxation, associated with cooperative molecular motions that involve simultaneous movements of electric dipoles due to the influence of neighboring groups; the β-process, which involves partially cooperative motions; and the γ-process, related to non-cooperative and highly localized motions where dipoles can move without interference from adjacent segments or dipoles [39,40]. Above the α-relaxation, additional phenomena such as the ρ-relaxation (associated with space charge or conductivity relaxation) and MWS relaxation may appear, depending on the polymer structure and the dielectric process involved.

Relaxation processes governed by small-scale relaxing entities (non-cooperative motions) generally follow an Arrhenius-type behavior expressed by Equation (17):

(17) $$\tau =\tau_{0}\exp\left(\frac{E_{a}}{k_{B}T}\right)$$

where $E_{a}$ is the activation energy, $k_{B}$ is the Boltzmann’s constant, T is the absolute temperature, and $\tau_{0}$ is a pre-exponential factor, typically ranging between 10^−16^ and 10^−13^ s. The lower value corresponds to entropic contributions, and the higher value corresponds to atomic frequency vibrations.

For the α-relaxation in amorphous and semicrystalline polymers, deviations from linear Arrhenius behavior are often observed. As the temperature decreases, fewer structural units have both a translational degree of freedom and an available neighboring site to move into. Experimentally, this manifests as a sharp increase in the relaxation time and a noticeable deviation from the Arrhenius law. To account for this behavior, two main theoretical frameworks have been proposed: free volume theory and the Adam–Gibbs theory. The most commonly used empirical expression to describe the temperature dependence of relaxation time is the Vogel–Fulcher–Tammann (VFT) equation:

(18) $$\tau =\tau_{0}\exp\left(\frac{B}{T-T_{0}}\right)$$

Here, B is a constant related to the activation energy, and $T_{0}$ is a temperature below which τ→∞. For the α-relaxation, it is accepted that molecular rearrangements of this type are no longer possible below $T_{0}$. However, the VFT equation does not clearly relate the parameters B and $T_{0}$ to the molecular dynamics of the relaxation process.

To bridge this gap, Matsuoka developed the cooperativity theory, based on the Adam–Gibbs model [41,42]. In this theory, the system is viewed as an ensemble of subsystems, where molecular rearrangements can only occur if all entities within a subsystem relax simultaneously (cooperative motion). The probability of transition between configurations depends mainly on the size of the smallest subsystem capable of transitioning without requiring the cooperative movement of neighboring regions.

Such a cooperatively rearranging region is defined as a subsystem that, due to sufficient thermal fluctuation, can switch from one configuration to another. The number of structural units within this region is denoted as Z. Assuming the independence of subsystems, a relationship is established between the configurational entropy of the macroscopic system and that of a subsystem with Z units:

(19) $$\frac{S_{ss}}{S_{M}}=\frac{Z}{N_{A}}$$

where $S_{ss}$ is the configurational entropy of the subsystem, $S_{M}$ is that of the entire system, and $N_{A}$ is the Avogadro’s number. A lower bound of entropy $S_{ss}^{*}$ exists, corresponding to a critical size $Z^{*}$:

(20) $$\frac{S_{ss}^{*}}{S_{M}}=\frac{Z^{*}}{N_{A}}$$

The apparent activation energy is then expressed as follows:

(21) $$∆E=\left(∆\mu \right)Z^{*}$$

Here, ∆μ represents the activation energy of an elementary molecular motion. According to the Maxwell–Boltzmann equipartition principle, the transition probability becomes

(22) $$P\propto \exp\left(-\frac{\left(∆\mu \right)Z^{*}}{k_{B}T}\right)$$

In the classical theory of rate processes, it is established that the rate constant is proportional to the probability P. The time constant for this transition is the relaxation time τ proportional to $P^{-1}$; consequently, the relaxation time is defined by the following equation:

(23) $$\tau =\tau_{0}\exp\left(\frac{\left(∆\mu \right)Z^{*}}{k_{B}T}\right)=\tau_{0}{\left[\exp\left(\frac{∆\mu }{k_{B}T}\right)\right]}^{Z^{*}}$$

When $Z^{*}=1$, the cooperatively rearranging region consists of a single unit, meaning all motions are independent, and the Arrhenius behavior is recovered. Several studies suggest that, for cooperative relaxation (such as α-relaxation), the relaxation time follows a power-law relationship [8]:

(24) $$\tau_{coop}=\tau_{0}{\left(\frac{\tau }{\tau_{0}}\right)}^{Z^{*}}$$

This can be derived from Equation (23), considering that τ corresponds to the Arrhenius-type relaxation time of the elementary molecular motions that collectively produce the cooperative motion. The exponent $Z^{*}$ changes with the temperature and can be estimated experimentally as follows:

(25) $$Z^{*}=\frac{T}{T-T_{0}}\cdot \frac{T^{*}-T_{0}}{T^{*}}\;\;$$

Here, $T_{0}\leq T\leq T^{*}$, where $T^{*}$ is a temperature above which $Z^{*}=1$. For fully amorphous polymers, typically, we obtain $T^{*}\approx 1.3T_{g}$, while in semicrystalline polymers, it may be associated with the melting temperature $T_{m}$. For the α-relaxation, the relationship has been reported as $T_{0}\approx T_{g}-50$ K [8,43].

## 3. Testing the Response of the Electrical Fractional Model (EFM)

**Figure 4:**
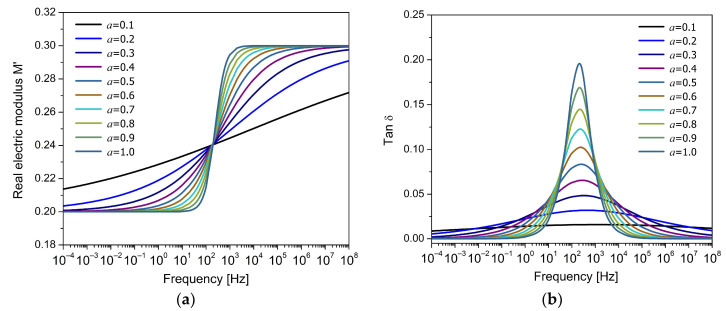
Frequency response of (**a**) the real part of the electric modulus ($M^{\prime }$) and (**b**) the loss factor (tanδ) for the FDM model, for different values of the fractional parameter a.

Figure 4a shows the variation in the real component of $M^{*}$, while Figure 4b presents the corresponding to tanδ, both as a function of frequency for different values of the fractional order a. The remaining parameters were selected heuristically in order to represent the typical response observed in polymeric materials. In Figure 4a, it is observed that the electric modulus increases with frequency, in contrast to the behavior of dielectric permittivity, which decreases with frequency. The theoretical curves intersect around the characteristic time of the relaxation process, with the fractional order controlling the shape of the curve, resulting in a symmetrical response. In Figure 4b, it is observed that the fractional order mainly controls the height and width of the dielectric loss peak. For low values of a (e.g., a=0.1), the peak is broad and shallow, indicating a strong dispersion in the relaxation times and a very slow relaxation process. As the fractional order increases, the peak becomes progressively taller and narrower, reaching its sharpest shape at a=1, corresponding to the classical Debye model. This can be interpreted as a change in the system’s ability to store and dissipate energy on different time scales. With a≪1, long-range memory effects and wide distribution of relaxation times are introduced, and with a≈1, the system responds more immediately to the applied field, concentrating the loss in a narrow frequency range.

**Figure 5:**
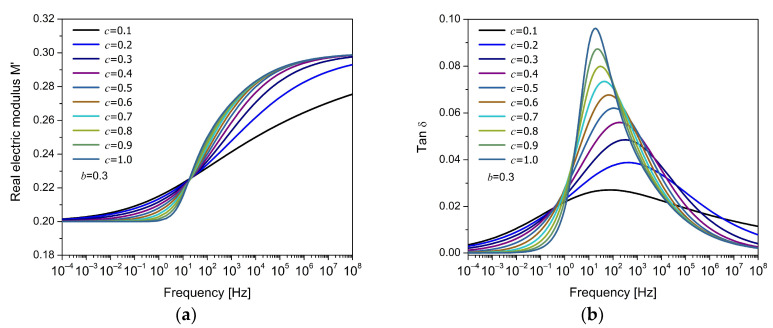
Frequency response of (**a**) the real part of the electric modulus ($M^{\prime }$) and (**b**) the loss factor (tanδ) for the FDM2 model, for different values of the fractional parameter c, with fixed b= 0.3.

Figure 5a,b show the variation in the electric modulus M′ and tanδ, respectively, as a function of frequency for different values of the fractional parameter c, holding constant the parameter b=0.3. This model is the FDM2, where the parameters b and c correspond to fractional orders that modify the response of the dielectric system. In all cases, a single relaxation peak is observed, characteristic of a Debye-type response. However, the shape of the peak changes significantly with the value of c. For low values of c (e.g., c=0.1), the peak is broad, low-intensity, and symmetrical, indicating a dispersed response and a broad relaxation time distribution. As c→1, the peak becomes more pronounced, narrower, and asymmetrical, concentrating in a more limited frequency region. This suggests a different molecular mobility around the peak (at low and high frequencies), which is strongly influenced by the fractional orders. When b=c=1, the response tends to the classical Debye behavior. Small values of c reflect greater temporal dispersion in the relaxation mechanisms, possibly associated with structural disorder or cooperativity between electric dipoles. In contrast, values close to unity indicate that relaxation occurs more uniformly and with less influence from these effects. It is worth noting that the parameter b=0.3, when held constant, also affects the peak width, and its low value implies a broader base in all cases.

**Figure 6:**
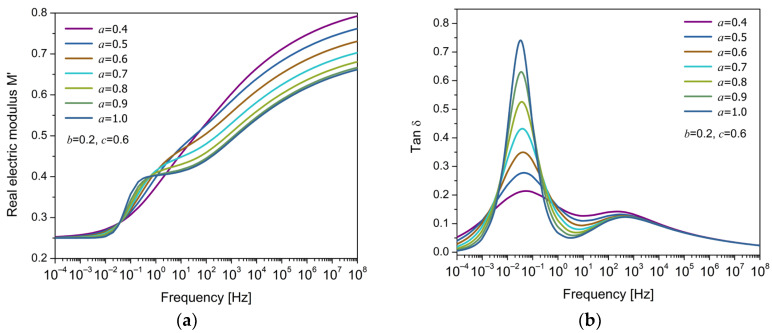
Frequency response of (**a**) the real part of the electric modulus ($M^{\prime }$) and (**b**) the loss factor (tanδ) for different values of the fractional parameter a, with fixed values b= 0.2 and c= 0.6.

From this analysis, it can be concluded that the Debye model with one or two cap-resistors is only capable of modeling a single dielectric relaxation process, yielding either symmetric or asymmetric responses depending on the fractional orders, which influence the molecular mobility of the electric charge carriers involved in the relaxation. Then, it is proposed to use the EFM to capture two distinct relaxation phenomena: α-relaxation and MWS. Previous studies by Alcoutlabi, Martínez-Vega, and Reyes-Melo et al. proposed the use of equivalent arrangements to take into account two and three mechanical relaxation models (considering the $E^{*}$ determined using DMA) and the dielectric relaxation processes (permittivity approach) that contemplate the main relaxation and secondary relaxations in amorphous and semicrystalline polymers [7,38,44,45]. In this work, Equation (16) is used to simultaneously model two electrical relaxation phenomena. The isothermal response is shown in Figure 6a, where two distinct steps are observed in the real part of the electric modulus $M^{\prime }$, corresponding to the relaxation phenomena involved. In Figure 6b, the loss factor (tanδ) exhibits two peaks, which are associated with MWS and α-relaxation, from lower to higher frequency. The fractional parameter a play a significant effect in the development of the relaxation behavior, particularly in the way partial energy dissipation is distributed across the frequency spectrum.

**Figure 7:**
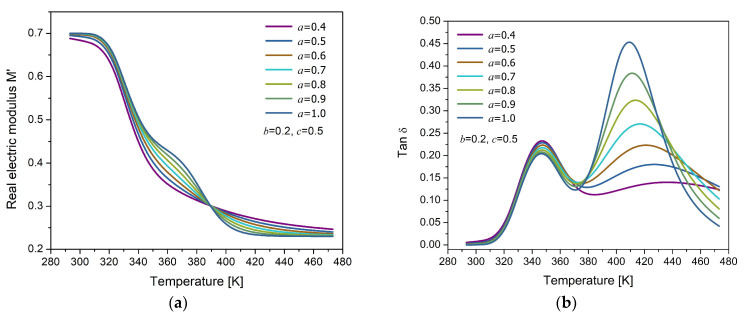
Temperature response of (**a**) the real part of the electric modulus ($M^{\prime }$) and (**b**) the loss factor (tanδ) for different values of the fractional parameter a, with fixed values b= 0.2 and c= 0.5.

The isochronous response for $M^{*}$ in the temperature domain is presented in Figure 7a. The model parameters were heuristically selected, with the Arrhenius equation (Equation (17)) applied to describe the MWS relaxation and the Matsuoka model (Equation (23)) used for the α-relaxation. Two distinct relaxation phenomena can be observed in Figure 7b: one at lower temperatures, associated with the primary relaxation of the polymer, representing the electrical manifestation of the glass transition, and another at higher temperatures, typically attributed to MWS interfacial polarization. The proximity between the two relaxation phenomena is due to the similar values of their activation energies. The influence of the fractional order is reflected in the modification of the relaxation dynamics; as the order increases, the MWS peak becomes more pronounced. This behavior suggests enhanced energy dissipation, which can be interpreted as a stronger conductive contribution to the dielectric response.

## 4. Comparison Between Theoretical Predictions and Experimental Data

### 4.1. Electrical Fractional Model in the Frequency Domain: Case Studies on PEI and PVC

In this section, we analyze three amorphous polymers extracted from the literature to validate the applicability of the proposed electrical fractional model (EFM). While the data for polyetherimide (PEI) and polyvinyl chloride (PVC) were extracted from peer-reviewed literature [16,46], the experimental results for polyvinyl butyral (PVB) were experimentally acquired by the authors using DEA, as detailed in reference [47]. The inclusion of both external and original datasets allows us to assess the generality of the model across different polymers and measurement conditions. The fitting was performed in the frequency domain, where the real and imaginary parts of the electric modulus were adjusted using the model.

**Figure 8:**
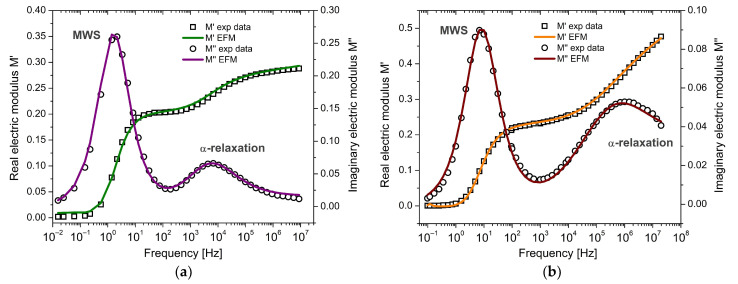
Frequency response of the complex electric modulus ($M^{\prime }$ and $M^{\prime \prime }$) for (**a**) PEI and (**b**) PVC: comparison between experimental data and the EFM model.

The first case corresponds to the work of Mudarra et al. [16], who investigated the relaxational behavior of PEI associated with their conductivity and dipolar relaxation. PEI is a high-performance amorphous thermoplastic with a relatively high glass transition temperature (*Tg*~493 K). Dielectric data were obtained using DEA, measuring the real and imaginary parts of the complex permittivity over a frequency range from 0.01 Hz to 1 MHz, with five frequency points per decade. Figure 8a displays the experimental dielectric response of PEI at 523 K, showing a low-frequency peak related to conductivity effects. The real and imaginary components of the electric modulus, $M^{*}$, were simultaneously fitted using the complete expression given in Equation (16), which incorporates both MWS and α-relaxation contributions. The electric modulus formalism inherently reduces the impact of low-frequency conduction. In particular, the imaginary part ($M^{\prime \prime }$) suppresses electrode polarization and dc-conductivity spurious effects, allowing the model to emphasize intrinsic bulk relaxation processes. This explains the good agreement observed in Figure 8a, even in the presence of moderate conductive behavior.

A second case study was selected from the work of Fahmy et al. [46], who reported the dielectric behavior of polyvinyl chloride (PVC), a widely used and cost-effective engineering thermoplastic. Measurements were carried out over a wide frequency range (0.1 Hz to 20 MHz). Figure 8b shows the experimental and modeled behavior of the electric modulus at 383 K. The real part $M^{\prime }$ increases non-linearly with frequency and reaches a plateau at higher frequencies. The imaginary part $M^{\prime \prime }$ reveals two distinct relaxation peaks: one at low frequencies attributed to interfacial polarization (MWS) and another at higher frequencies corresponding to α-relaxation, associated with cooperative segmental motion and C–Cl dipole reorientation in PVC [46]. Although interfacial polarization is typically associated with heterogeneous systems, it may also occur in nominally homogeneous polymers such as PVC due to internal dielectric inhomogeneities. These may include morphological irregularities, amorphous–crystalline interfaces, or residual processing agents. In PVC, the combination of high dielectric permittivity and low conductivity enhances space charge accumulation at such internal discontinuities, resulting in a pronounced low-frequency MWS-type relaxation.

A total of ten parameters were used in the model fitting: for MWS relaxation, $M_{1\infty }$, $M_{1s}$, $\tau_{a}$,a and for α-relaxation, $M_{2\infty }$, $M_{2s}$, $\tau_{b}$, $\tau_{c}$, b, c. These parameters correspond to the analytical expressions described in Equation (16). The results show that each contribution dominates a distinct frequency region with minimal overlap and that both are necessary to describe the full dielectric response accurately. Figure 8 shows the agreement between the experimental data and the fitted model. A summary of the optimized parameter values is provided in Table 1.

### 4.2. Electrical Fractional Model in the Temperature Domain: A Case Study on PVB

In this section, we apply the proposed electrical fractional model (EFM) to describe the temperature-dependent dielectric response of polyvinyl butyral (PVB), using literature data to evaluate the model’s ability to capture thermally relaxation processes. Specifically, we investigate whether the EFM can distinguish between interfacial polarization (MWS effect) and α-relaxation by analyzing the evolution of the electric modulus with temperature, under the framework of the cooperativity theory.

**Figure 9:**
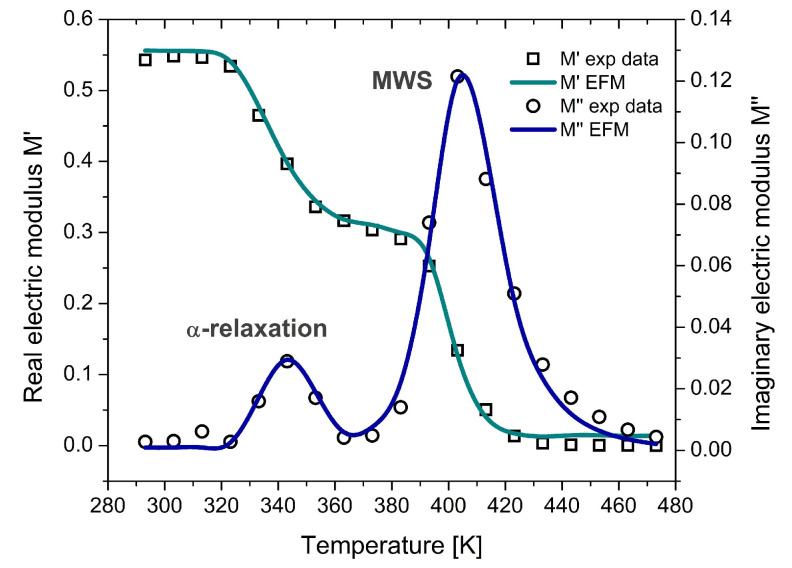
Temperature-dependent response of the complex electric modulus ($M^{\prime }$ and $M^{\prime \prime }$) for PVB, showing the α-relaxation and MWS relaxation modeled using fractional calculus.

PVB is an amorphous copolymer widely used in laminated safety glass due to its viscoelasticity, mechanical damping, and optical clarity. The experimental dataset, reported by Puente-Córdova et al. [47], includes dielectric measurements performed via dynamic electrical analysis across a frequency range from 100 Hz to 1 MHz and temperatures from 293 K to 473 K. Figure 9 presents the isochronal representation at 100 Hz. The experimental response fitted with the EFM reveals two well-defined peaks: the first, near 343 K, is attributed to the α-relaxation, characterized by cooperative segmental motion of dipoles; the second, near 403 K, corresponds to interfacial polarization, associated with the release and long-range motion of trapped charge carriers. These observations confirm the model’s capacity to discriminate between relaxation processes that occur at different spatial and thermal scales.

At lower temperatures, α-relaxation dominates, reflecting short-range, localized molecular reorientations. As temperature increases, mobility transitions toward more extended segmental motion, enabling charge displacement across distances comparable to the size of a PVB repeat unit. The MWS contribution, in contrast, becomes prominent at higher temperatures, where long-range ionic mobility is thermally activated. This behavior indicates a shift from localized to delocalized dynamics within the polymer matrix. To capture these thermally induced changes, the EFM is fitted in the temperature domain using two different temperature-dependence models appropriate for each relaxation mechanism. Specifically, Equation (23) is employed to describe the temperature dependence of the relaxation times associated with α-relaxation, as this process is strongly cooperative and deviates from Arrhenius behavior near the glass transition. The size of the cooperative rearranging regions can be estimated using the cooperativity theory, providing a molecular-level interpretation of the α-relaxation mechanism in PVB.

In contrast, the interfacial polarization (MWS) relaxation is modeled using an Arrhenius-type expression, which is more suitable for thermally activated, non-cooperative processes such as charge hopping and ion migration. This approach allows for a meaningful interpretation of the MWS-related parameters in terms of activation energy and conductivity behavior above *Tg*. The model captures the main features of the experimental data across the studied temperature range. The fitted parameters are summarized in Table 2, and the modeled curve is plotted alongside experimental results in Figure 9, validating the EFM’s ability to describe complex dielectric phenomena in thermally activated, amorphous polymer systems. It is worth noting that PVB is a copolymer composed of vinyl butyral, vinyl alcohol, and vinyl acetate. The successful modeling of its dielectric relaxation using the EFM indicates that the model is applicable not only to homopolymers but also to copolymers, provided that the relaxation mechanisms are macroscopically well-defined, and the material exhibits an effectively homogeneous dielectric response.

### 4.3. Discussion About the Electrical Fractional Model

The results presented in Figure 8 and Figure 9, along with the corresponding fitted parameters summarized in Table 1 and Table 2, demonstrate that the proposed electrical fractional model (EFM) provides a coherent and consistent description of dielectric relaxation processes in both frequency and temperature domains. In the case of PEI and PVC (Table 1), the fitting procedure successfully distinguishes two main contributions: one associated with interfacial polarization (MWS) and another with α-relaxation. For both materials, the fractional order a is close to 0.9, indicating a relatively well-defined Debye-like behavior for the MWS contribution. However, the α-relaxation, modeled using two cap-resistors (orders b and c), exhibits broader and more complex dynamics. The low values of b (0.06–0.07) and intermediate values of c (0.52–0.67) reflect a highly dispersive process, with significant non-exponentiality and dynamic heterogeneity, especially at low frequencies.

This behavior is consistent with the expected dielectric response of amorphous polymers, where the cooperative motion of chain segments and dipoles results in a wide distribution of relaxation times. Furthermore, in the frequency domain, the time constants associated with α-relaxation ($\tau_{b}$, $\tau_{c}$) are shorter than those for MWS ($\tau_{a}$), reinforcing the interpretation that dipolar segmental motions are faster and more localized than interfacial polarization mechanisms.

Regarding the thermal analysis of PVB (Table 2), the EFM fitting confirms the coexistence of α-relaxation and interfacial polarization, with distinct peaks observed at approximately 343 K and 403 K, respectively. The parameter a is very close to 1, indicating that interfacial polarization in PVB at elevated temperatures behaves nearly resistively, reflecting a conductive response. This is also supported by the low values of the relaxation times ($\tau_{0a}$, $\tau_{0b}$, $\tau_{0c}$~10^−13^ s), which suggests that both processes are thermally stimulated and occur on fast time scales.

To accurately describe the temperature evolution of each process, the model combines two different formulations for relaxation times: an Arrhenius-type dependence for MWS, consistent with charge transport or hopping processes, and a cooperativity theory for the α-relaxation, which captures the non-Arrhenius behavior typical of cooperative segmental motions near the glass transition. The extracted parameters ($T_{0}$, $T^{*}$, $E_{a}$, $E_{b}$, and $E_{c}$) are physically meaningful and agree with trends reported for amorphous systems.

Overall, the model reproduces the main features of the experimental dielectric response for the three polymers used as examples. It captures the separation between short-range (α-relaxation) and long-range (MWS) polarization processes, the effect of frequency and temperature, and the non-exponential character of the relaxation. These results support the suitability of fractional-order modeling approaches, particularly those based on electric modulus formalism, to describe the dielectric behavior with cooperative dynamics and interfacial effects. In addition to its application to polymeric materials—such as amorphous and semicrystalline polymers, blends, homopolymers, and copolymers—the EFM can also be extended to describe systems like suspensions, including colloidal dispersions, nanoparticle-filled polymers, and emulsions, which exhibit broad and overlapping dielectric relaxations [48,49]. In such systems, interfacial polarization, diffuse double-layer effects, and ion migration can lead to complex dielectric spectra that are often better captured using fractional calculus. This approach, inherently capable of capturing non-Debye and power-law dynamics, is particularly well-suited for modeling complex dielectric behavior.

## 5. Conclusions

This work presents an electrical fractional model (EFM) based on the electric modulus formalism and fractional calculus, capable of simultaneously describing two dielectric relaxation phenomena in polymers: the main α-relaxation and interfacial polarization (MWS effect). By incorporating cap-resistor elements into an extended Debye model, the proposal successfully captures the complex, non-Debye behavior typically observed in amorphous polymer systems. The model was validated in both the frequency and temperature domains using literature data for PEI, PVC, and PVB. The EFM proved effective in reproducing two distinct relaxation contributions across a wide range of conditions. In particular, the fractional orders obtained for the MWS component (a≈ 0.90–0.99) consistently reflected a near-resistive response and low dispersion, while those for the α-relaxation (b, c ≈ 0.06–0.32) indicated higher dispersity and dynamic heterogeneity, in agreement with the cooperative nature of dipolar segmental motion. The relaxation times associated with α-relaxation ($\tau_{b}$, $\tau_{c}$) were found to be shorter than those for MWS ($\tau_{a}$), supporting the interpretation that α-processes involve faster, localized molecular motions. Additionally, the activation energies obtained from the temperature-dependent fits further differentiated the phenomena: higher values for MWS reflected the influence of conductive mechanisms, space charge accumulation, and trap release, while the lower energies of α-relaxation aligned with cooperative mobility near the glass transition. The use of distinct temperature-dependence expressions, namely the Matsuoka model for α-relaxation and the Arrhenius model for MWS, provided physically meaningful fits and helped separate the contributions in the electric modulus formalism. Overall, the EFM is a powerful tool for analyzing complex dielectric responses in polymers, with clear potential application to other systems exhibiting coupled or overlapping relaxation mechanisms.

## Figures and Tables

**Table 1:** Fitting parameters of the electrical fractional model in the frequency domain.

EFM Parameters	PEI	PVC
$M_{1s}$	0.17	0.17
$M_{1\infty }$	0.80	0.40
$\tau_{a}$ (s)	5.50 × 10^−1^	5.10 × 10^−2^
a	0.89	0.88
$M_{2s}$	0.60	0.22
$M_{2\infty }$	1.15	1.60
$\tau_{b}$ (s)	1.57 × 10^−3^	2.10 × 10^−5^
$\tau_{c}$ (s)	1.99 × 10^−5^	6.98 × 10^−7^
b	0.06	0.07
c	0.67	0.52

**Table 2:** Fitting parameters of the electrical fractional model in the temperature domain.

EFM Parameters	PVB
$M_{1s}$	0.30
$M_{1\infty }$	0.90
$E_{a}$(eV)	0.75
$\tau_{0a}$(s)	1.40 × 10^−13^
a	0.99
$M_{2s}$	0.025
$M_{2\infty }$	0.30
$E_{b}$(eV)	0.46
$E_{c}$(eV)	0.51
$T^{*}$(K)	445
$T_{0}$(K)	285
$\tau_{0b}$(s)	1.20 × 10^−13^
$\tau_{0c}$(s)	1.10 × 10^−13^
b	0.25
c	0.32

## Data Availability

The original contributions presented in this study are included in the article. Further inquiries can be directed to the corresponding authors.
